# Supplementary material for: An exploration of under-registration of chronic kidney disease stages 3–5 in Belgian general practices using logistic regression
Source: PLoS One. 2022 Dec 22;17(12):e0279291. doi: 10.1371/journal.pone.0279291 (PMC9778624; doi:10.1371/journal.pone.0279291)
Supplement: S1 Table — (DOCX) [file pone.0279291.s001.docx]

**Supporting information**

**S1 Table. Missing data.**

| **Variable** | **Missingness registered CKD** | **Missingness unregistered CKD** | **P-value** |
| --- | --- | --- | --- |
| Total patients | 3375 | 7176 |  |
| **Demographics at baseline** |  |  |  |
| Males, n (%) | complete | complete | 0.001 |
| Females, n (%) | complete | complete | 0.001 |
| Mean Age (years) (sd) | complete | complete | 0.001 |
| 45-65 years, n (%) | complete | complete | 0.001 |
| 65-75 years, n (%) | complete | complete | 0.001 |
| 75-plus years, n (%) | complete | complete | 0.001 |
| Mean Height (cm) (sd) | 94.6 % | 96.2 % | 0.001 |
| Mean Weight (kg) (sd) | 58.2 % | 64.7 % | 0.001 |
| Mean BMI (kg/m^2^) (sd) | 70.2 % | 73.4 % | 0.001 |
| **Laboratory tests at baseline** |  |  |  |
| Mean hemoglobin (sd) | 1.9 % | 1.6 % | 1.9 % |
| hemoglobin < 8 g/100mL, n (%) | complete | complete | complete |
| hemoglobin 8-10 g/100mL, n (%) | complete | complete | complete |
| hemoglobin 10-12 g/100mL, n (%) | complete | complete | complete |
| hemoglobin 12-plus g/100mL, n (%) | complete | complete | complete |
| Mean hematocrit (%) (sd) | 3.5 % | 2.5 % | 3.5 % |
| Mean egfr (mL/min/1,73 m2) (sd) | 0% | 0% | 0% |
| Mean potassium (sd) | 12.4 % | 18.2 % | 12.4 % |
| potassium <5.0 mmol/L, n (%) | complete | complete | complete |
| potassium 5.0-5.5 mmol/L, n (%) | complete | complete | complete |
| potassium 5.5-6.0 mmol/L, n (%) | complete | complete | complete |
| potassium 6.0-6.5 mmol/L, n (%) | complete | complete | complete |
| potassium 6.5-plus mmol/L, n (%) | complete | complete | complete |
| Mean uACR (mg/g creatinine) (sd) | 91.9 % | 94.2 % | 91.9 % |
| Mean uPCR (mg/g creatinine) (sd) | 98.1 % | 98% | 98.1 % |
| Mean uric_acid (mg/dL) (sd) | 24.8 % | 22.9 % | 24.8 % |
| Mean total cholesterol (mg/dL) (sd) | 19.6 % | 18.4 % | 19.6 % |
| Mean hdl cholesterol (mg/dL) (sd) | 21.7 % | 21.3 % | 21.7 % |
| Mean ldl cholesterol (mg/dL) (sd) | 50.4 % | 47.6 % | 50.4 % |
| Mean triglyceriden (mg/dL) (sd) | 21.6 % | 20.9 % | 21.6 % |
| **Prescriptions at baseline** |  |  |  |
| ACE inhibitors, n (%) | complete | complete | complete |
| ARB drugs, n (%) | complete | complete | complete |
| ARNI drugs, n (%) | complete | complete | complete |
| MRA drugs, n (%) | complete | complete | complete |
| Loop diuretics, n (%) | complete | complete | complete |
| Beta blockers, n (%) | complete | complete | complete |
| Thiazide diuretics, n (%) | complete | complete | complete |
| Alpha blockers, n (%) | complete | complete | complete |
| Metformins, n (%) | complete | complete | complete |
| Sulfonylurea, n (%) | complete | complete | complete |
| DDP-4 inhibitors, n (%) | complete | complete | complete |
| GLP-1RA, n (%) | complete | complete | complete |
| Insulins, n (%) | complete | complete | complete |
| Other OAD, n (%) | complete | complete | complete |
| SGLT-2 inhibitors, n (%) | complete | complete | complete |
| Lipid lowering drugs, n (%) | complete | complete | complete |
| Platelet aggregation inhibitors, n (%) | complete | complete | complete |
| Anticoagulants, n (%) | complete | complete | complete |
| **Comorbidities at baseline** |  |  |  |
| Myocardial infarction (ICPC2 K75), n (%) | complete | complete | complete |
| Angina pectoris (ICPC2 K74), n (%) | complete | complete | complete |
| Stroke (ICPC2 K90), n (%) | complete | complete | complete |
| Coronary atherosclerosis (ICPC2 K76), n (%) | complete | complete | complete |
| Peripheral vascular disease (ICPC2 K92), n (%) | complete | complete | complete |
| Heart failure (ICPC2 K77), n (%) | complete | complete | complete |
| Atrial fibrillation (ICPC2 K78), n (%) | complete | complete | complete |
| Hypertension (ICPC2 K86), n (%) | complete | complete | complete |
| Hypertensive kidney failure (ICPC2 K87), n (%) | complete | complete | complete |
| Type 1 Diabetes (ICPC2 T89), n (%) | complete | complete | complete |
| Type 2 Diabetes (ICPC2 T90), n (%) | complete | complete | complete |
| Hyperkalemia (ICPC2 T99), n (%) | complete | complete | complete |
| Glomerulonephritis (ICPC2U88), n (%) | complete | complete | complete |
| Unspecified kidney disease (ICPC2 U99), n (%) | complete | complete | complete |
| Poly cystic Kidney disease (ICPC2 U85), n (%) | complete | complete | complete |
| Systemic Lupus erythematosus (ICPC2 L99), n (%) | complete | complete | complete |
| Gout (ICPC2 T92), n (%) | complete | complete | complete |
| Covid-19 infection (virus identified) (ICPCC 2 A77), n (%) | complete | complete | complete |
| Covid-19 infection (virus not identified) (ICPC2 R80), n (%) | complete | complete | complete |
