# Supplementary material for: An exploration of under-registration of chronic kidney disease stages 3–5 in Belgian general practices using logistic regression
Source: PLoS One. 2022 Dec 22;17(12):e0279291. doi: 10.1371/journal.pone.0279291 (PMC9778624; doi:10.1371/journal.pone.0279291)
Supplement: Attachment — Submitted filename: Response to Reviewers.docx [file pone.0279291.s002.docx]

Leuven,

25/08/2022

Dear Editor,

Dear reviewers,

Please find enclosed a revised version of our manuscript entitled “An exploration of under-registration of chronic kidney disease in Belgian general practices using logistic regression”.

We gratefully thank you for considering our manuscript for publication and providing these important comments. We have studied your comments and suggestions carefully and we have adapted the manuscript accordingly. We truly believe that this process has improved the quality of the manuscript substantially.

We made some adjustments based on the editor’s comments:

1. **Please ensure that your manuscript meets PLOS ONE's style requirements, including those for file naming. The PLOS ONE style templates can be found at
   https://journals.plos.org/plosone/s/file?id=wjVg/PLOSOne_formatting_sample_main_body.pdf and 
   https://journals.plos.org/plosone/s/file?id=ba62/PLOSOne_formatting_sample_title_authors_affiliations.pdf**

*File naming was adjusted according to PLOS ONE’s requirements.*

2. **Please amend your current ethics statement to address the following concerns:
   a) Did participants provide their written or verbal informed consent to participate in this study?
   b) If consent was verbal, please explain i) why written consent was not obtained, ii) how you documented participant consent, and iii) whether the ethics committees/IRB approved this consent procedure.**

*The INTEGO procedure was approved by a written consent by the ethical review board of the Medical School of KU Leuven (N° ML 1723) and by the Belgian Privacy Commission (no SCSZG/13/079). Written consent was obtained from every of the participating Intego primary care practices.*

3. **Please note that PLOS ONE has specific guidelines on code sharing for submissions in which author-generated code underpins the findings in the manuscript. In these cases, all author-generated code must be made available without restrictions upon publication of the work. Please review our guidelines at https://journals.plos.org/plosone/s/materials-and-software-sharing#loc-sharing-code and ensure that your code is shared in a way that follows best practice and facilitates reproducibility and reuse.**

*The code was attached in a separate word file.*

4. **Thank you for stating the following in your Competing Interests section:
   "No authors have competing interests."
    
   Please complete your Competing Interests on the online submission form to state any Competing Interests. If you have no competing interests, please state ""****The authors have declared that no competing interests exist."", as detailed online in our guide for authors at http://journals.plos.org/plosone/s/submit-now 
    This information should be included in your cover letter; we will change the online submission form on your behalf.**

*We have no Competing Interests. The following statement was added in the cover letter: "The authors have declared that no competing interests exist."*

5. **In your Data Availability statement, you have not specified where the minimal data set underlying the results described in your manuscript can be found. PLOS defines a study's minimal data set as the underlying data used to reach the conclusions drawn in the manuscript and any additional data required to replicate the reported study findings in their entirety. All PLOS journals require that the minimal data set be made fully available. For more information about our data policy, please see http://journals.plos.org/plosone/s/data-availability.**

**Upon re-submitting your revised manuscript, please upload your study’s minimal underlying data set as either Supporting Information files or to a stable, public repository and include the relevant URLs, DOIs, or accession numbers within your revised cover letter. For a list of acceptable repositories, please see http://journals.plos.org/plosone/s/data-availability#loc-recommended-repositories. Any potentially identifying patient information must be fully anonymized.**

**Important: If there are ethical or legal restrictions to sharing your data publicly, please explain these restrictions in detail. Please see our guidelines for more information on what we consider unacceptable restrictions to publicly sharing data: http://journals.plos.org/plosone/s/data-availability#loc-unacceptable-data-access-restrictions. Note that it is not acceptable for the authors to be the sole named individuals responsible for ensuring data access.**

**We will update your Data Availability statement to reflect the information you provide in your cover letter.**

*As stated when submitting, it is not possible to make a minimum dataset publicly available, but data requests can be made to the Data Protection Officer (Sciensano) .*

*The data set necessary to validate our analysis contains identifying patient-level data which cannot be suitably de-identified or aggregated and can therefore only be accessed inside a monitored analysis environment. These restrictions were imposed by the Belgian National Information Security Committee: section Social Security and Health:*

*°Websites (only FR and NL):https://ehealth.fgov.be/ehealthplatform/nl/informatieveiligheidscomite https://ehealth.fgov.be/ehealthplatform/fr/comite-de-securite-de-linformation oMain deliberations (only FR and NL):*

*- Beraadslaging nr. 15/009 van 17 februari 2015 betreffende de generieke methode voor de uitwisseling van gecodeerde persoonsgegevens die de gezondheid betreffen in het kader van healthdata.be en healthstat.be  (https://ehealth.fgov.be/ehealthplatform/file/view/23acd078e8a4cb025e216b6d24906ae 480b57cf8?filename=15-009-n023-healthdata_be.pdf)*

*- Délibération n° 15/009 du 17 février 2015 relative à la méthode générique d'échange de données à caractère personnel codées relatives à la santé, dans le cadre de healthdata.be et healthstat.be. (https://ehealth.fgov.be/ehealthplatform/file/view/ea116a489cc473f8174c8559dd060b4f aa3ff651?filename=15-009-f023-healthdata_be.pdf)*

*° INTEGO deliberations (only FR and NL):*

*- Beraadslaging nr. 13/026 van 19 maart 2013, laatst gewijzigd op 7 april 2020, met betrekking tot de mededeling van gepseudonimiseerde persoonsgegevens die de gezondheid betreffen, via het healthdata.be platform, in het kader van de samenstelling, het gebruik en de ter beschikking stelling van een register voor epidemiologisch onderzoek (Intego). è https://ehealth.fgov.be/ehealthplatform/file/view/AXGB3PooVF2eEzQeBL5P?filename= 13-026-n162-INTEGO%20Covid19-gewijzigd%20op%207%20april%202020.pdf*

*- Délibération n° 13/026 du 19 mars 2013, modifiée en dernier lieu le 7 avril 2020, relative à la communication de données à caractère personnel pseudonymisées relatives à la santé, via la plateforme healthdata.be, dans le cadre de la constitution, de l'utilisation et de la mise à la disposition d'un registre de recherches en épidémiologie (Intego).  (https://ehealth.fgov.be/ehealthplatform/file/view/AXGB2dtUmTlaOSp4Nmk3?filename= 13-026-f162-INTEGO%20Covid19-modifi%C3%A9e%20le%207%20avril%202020.pdf)*

*Data requests may be sent to Mr. Roel Heijlen, Data Protection Officer healthdata.be (Sciensano), Roel.Heijlen@sciensano.be, Rue Juliette Wytsmanstraat 14, 1050 Brussels. Interested researchers will need to provide their name, first name, professional organization name, email address, mobile number and the database of interest to request access.*

6. **Please upload a copy of Supporting Information Table 1 which you refer to in your text on page 18.**

*Supporting Information Table 1 was added.*

We also made some adjustments based on the reviewer’s comments:

1. **Page 6, lines 108, Methods: In order to correctly define CKD, the calculation of eGFR should be standardized. The authors did not mention the equation that calculated eGFR. According to the KDIGO (as the reference No. 2 of this manuscript) or the KDOQI guidelines,** **eGFR should be calculated using the MDRD or 2009 CKD-EPI equation. A newer 2021 CKD-EPI equation has also been suggested recently.**

*Thank you for this comment. Not all eGFR were calculated by the same equation due to the design of the database, which collects data from practices throughout Flanders. However, we selected these either based on MDRD or CKD-EPI. We added this in the manuscript.*

2. **Page 6, lines 108, Methods: the authors only included stages 3-5 CKD because they only include those who had an eGFR < 60 ml/min/1.73m2. This information should be more clearly described throughout the manuscript from the title, abstract, and manuscript text. Otherwise, it may cause misinterpretation that the study is providing information including the whole range of CKD patients.**

*Thank you for this suggestion. This was added in the title, abstract, introduction and conclusion.*

3. **Page 6, lines 111-112, Methods: The author used data since 2018, but did not mention the date for end of follow-up of the data.**

*We should have mentioned the date for end of follow-up of the data. This was 31/03/2021. It was added in the manuscript.*

4. **Page 7, lines 127-129, Methods: This section provides almost the same information as line 107-111.**

*Thank you for this comment. The inclusion criteria section was modified with reference to the study population section.*

5. **Page 8, lines 145-146, Methods: This description does not seem to be the calculation of "median time", should be explained more clearly. In addition, this sentence seems to provide very similar information as line 147-149.**

- *Lines 145-146: This was indeed a description of the diagnostic delay and not the median time. This has been adjusted and was described more clearly.*
- *Lines 147-149: Similar information was deleted.*

6. **Page 8, lines 160-161, Methods: The author did not mention the process of model selection for the multivariate logistic regression, and how to decide the final model as they presented in the Results section. Since the INTEGO database provided abundant information and covariates, how did author decide the inclusion and exclusion of these covariates? In addition, some of the covariates included in the multivariate model were not provided in the baseline descriptive analyses (Table 3) and some of the covariates did not have a clear definition (ex. did the covariate of diabetes include both type 1 & 2 diabetes? Did the covariate of hypertension included hypertensive kidney failure?)**

*Thank you for this fair comments. We used covariates based on clinical expertise after consultation with experts in the field.*

*Smoking and eGFR changes were left out in the submitted version. We now added them in table 3.*

*Diabetes included both type 1 & 2, hypertension did not include hypertensive kidney failure. Lines 248-251: We added the ICPC2 codes to clarify the definition of the covariates.*

7. **Pages 8-9, lines 162-171, Methods: The imputation method for missing variables was not clearly described. The imputation procedure seemed to be based on unpublished data (Reference No. 22), and the detailed imputation method should be clearly and comprehensively described in the current manuscript unless this imputation method has been published. How many covariates has been imputed? What is the percentage of missing data in the study cohort? A comparison before and after imputation should be provided. The Supporting information (S1 Table. Missing data) was not provided with the submitted manuscript. In addition, what is an extra covariate?**

*The imputation method was indeed based on unpublished data. Only smoking was missing. Therefore, we imputed the smoking variable for the year 2018, using the method of Multiple Imputation as described by Rubin (1) and implemented in Rpackage mice by Van Buuren (2) using several auxiliary variables including age, gender, socioeconomic status, and relevant diagnoses and prescriptions. The percentage of missing data was added and a figure with results before and after multiple imputation was provided (figure 4 and 5). The supporting information was attached.*

(1) *Rubin DB. Multiple Imputation for Nonresponse in Surveys. New York: John Wiley and Sons; 2004.*
(2) *Van Buuren, S., & Groothuis-Oudshoorn, K. (2011). mice: Multivariate imputation by chained equations in R. Journal of statistical software, 45, 1-67.*
8. **Page 11, lines 223-226, Results: There seemed to be difference between registered and unregistered groups for diuretics, lipid lowering drugs, platelet aggregation inhibitors, and anticoagulants.**

*This is a fair comment. There are indeed differences in percentage between registered and unregistered groups. The word choice was incorrect, so we adjusted it.*

9. **Page 11, lines 228-230, Results: the description regarding diagnosis code U99 provide duplicate information as in the Methods section (lines 117-120).**

*Thank you for this comment. We removed it in the method section, eliminating duplicate information.*

10. **Table 3: There were 309 (4.3%) of unregistered CKD patients who had a diagnosis code of "Unspecified kidney disease (ICPC2 U99)" at baseline which is confusing. Please explain.**

*As explained at line 231-235, patients with unspecified kidney disease are known to have a urinary tract disease other than renal insufficiency like overactive bladder, renal cyst,… Unfortunately, the ICPC2 code for chronic kidney disease is the same as the code for unspecified kidney disease, namely U99. Some of the patients with unspecified kidney disease also had decreased renal function, so they should also have a diagnostic code of CKD.*

Sincerely,

Ine Van den Wyngaert & co-authors
